# Editorial: Epithelial immune microenvironment and inflammatory skin diseases

**DOI:** 10.3389/fimmu.2024.1428209

**Published:** 2024-05-20

**Authors:** Zhongjian Chen, Xiaowei Xu, Yi Lu

**Affiliations:** ^1^Shanghai Skin Disease Hospital, School of Medicine, Tongji University, Shanghai, China; ^2^Abramson Cancer Center, Perelman School of Medicine, University of Pennsylvania, Philadelphia, PA, United States; ^3^Key Laboratory of Smart Drug Delivery of Ministry of Education (MOE), School of Pharmacy, Fudan University, Shanghai, China

**Keywords:** epithelial immune microenvironment, fibroblasts, histone modification, 5-aminolevulinic acid, epithelial-associated cDC2, glycolysis, acquired reactive perforating collagenosis, Traditional Chinese Medicine

The epithelial tissues constitute the outermost layer of our body, serving as a physical barrier to maintain tissue homeostasis. Immune events primarily take place in the epidermis and papillary dermis, collectively known as the epithelial immune microenvironment (EIME) (1, 2). Epithelial tissues function as immunological organs that protect the human body from pathogen invasion. Changes in the EIME in response to internal and external stimuli can trigger inflammatory responses, leading to the development of inflammatory skin diseases. Targeting the EIME presents a novel therapeutic approach for managing inflammatory skin conditions by modulating inflammatory processes. Unraveling disease-specific EIMEs facilitates the development of innovative therapies for inflammatory skin diseases.

Fibroblasts have been historically regarded as passive bystanders in the EIME. However, recent advances in single-cell technologies such as RNA sequencing and spatial transcriptomics have challenged this notion. Wang et al. have provided new insights into the role of fibroblasts in inflammation progression. In fibrotic skin diseases, fibroblasts become overactivated, resulting in excessive collagen accumulation and fibrous hyperproliferation. Additionally, activated fibroblasts communicate with other types of cells in the cutaneous EIME through the secretion of chemokines or proinflammatory substances. This intercellular crosstalk is significantly involved in the pathogenesis of both fibrotic and non-fibrotic inflammatory diseases, making fibroblasts the hallmark cells in these conditions. Targeting the activation of fibroblasts provides a promising therapeutic strategy for treating fibrotic skin diseases.

Zhang et al. conducted a comprehensive review on the relationship between histone modification and inflammatory skin diseases, as well as the corresponding therapeutic approaches. Acetylation and methylation were identified as the primary types of histone modification associated with inflammatory skin disease. It was observed that histone modification not only influences the polarization of macrophages but also impacts the differentiation and function of T cells, thereby promoting the pathogenesis and progression of inflammatory skin diseases. Short-chain fatty acids have emerged as inhibitors of histone deacetylase (HDAC), exhibiting antiproliferative and anti-inflammatory properties that make them promising novel therapeutics for inflammatory skin diseases. Furthermore, bromodomain and extraterminal protein inhibitors, which are involved in transcription following histone modification, may also hold potential for the treatment of inflammatory skin diseases.

Yan et al. utilized single-cell transcriptome profiling to investigate the EIME change in photoaged skin following 5-aminolevulinic acid photodynamic therapy. The analysis revealed that photoaged skin is characterized by increased cellular senescence in immune cells, decreased immune receptor activity, a reduced proportion of naive T cells, the impaired function of T cell ribosomal synthesis, and up-regulation of the G2M checkpoint. However, treatment with 5-aminolevulinic acid was found to partially reverse the immunosenescence and improve the immunosuppressive state, leading to rejuvenating effects that persisted for at least 6 months.

Lang et al. studied the microenvironmental cues and factors that regulate the differentiation of epithelial-associated cDC2 under inflammatory conditions. Inflammatory signals have been shown to drive the differentiation of human cDC2s into various cell subsets present in the psoriatic epidermis. Bone morphogenetic protein cooperates with canonical TGF-b1 signaling for inducing Axl^+^cDC2s from blood cDC2s, which further differentiate into Langerhans cells. Additionally, p38MAPK promoted the generation of RelB^+^cDC2s, being predominantly at dermal sites in the inflamed skin. These findings suggest a potential target for future immunotherapy in psoriasis.

Hyperproliferative keratinocytes are one of the pathophysiological hallmarks in the EIME of psoriatic lesions, potentially driven by increased glycolysis and elevated expression of glucose transporter 1. Shou et al. demonstrated elevated levels of glycolysis in psoriasis by analyzing the RNA seq database of 345,414 cells from different cohorts. They identified a novel subpopulation of keratinocytes expressing SLC2A1 and LDH1, which are associated with glycolysis. A set of 12 common transcriptome signatures were defined. A glycolysis score-based model was developed to predict the psoriasis treatment outcome, which was validated by Decision Curve Analysis.

Acquired reactive perforating collagenosis (ARPC) is a clinically challenging disease with an unclear pathogenesis, characterized by the extrusion of necrotic collagen through the epidermis. Liu et al. revealed the infiltration of CD3+ T-cells in the dermis and subcutaneous tissue of ARPC patients, along with a predominance of Th2 cells over Th1 cells and overexpression of IL-4 and IL-13 in ARPC lesions. The findings imply the involvement of type 2 inflammation in the pathogenesis of ARPC. Liu et al. also confirmed the safety and efficacy of Dupilumab in managing itching and reducing skin lesions in ARPC. Dupilumab represents a promising treatment option for ARPC patients who do not respond to conventional therapies.

Traditional Chinese Medicine provides a new remedy for skin diseases. Li et al. verified the therapeutic effect of Fufang Honghua Buji (FHB) granules on vitiligo and elucidated their molecular mechanisms using network pharmacology, molecular docking, and molecular dynamics simulation. Luteolin, quercetin, and wogonin were identified as the main active components in vitiligo treatment, with the JAK-STAT pathway being the primary target. FHB was found to suppress genes involved in JAK-STAT pathway, reduce inflammation, and promote melanogenesis.

The newly developed single-cell technologies are renewing and deepening our acknowledgments to the EIME. Promising remedies for inflammatory skin diseases by targeting the EIME are emerging.

## Author contributions

ZC: Writing – original draft. XX: Writing – review & editing. YL: Writing – review & editing, Writing – original draft.
